# Breastfeeding and maternal cardiovascular risk factors and outcomes: A systematic review

**DOI:** 10.1371/journal.pone.0187923

**Published:** 2017-11-29

**Authors:** Binh Nguyen, Kai Jin, Ding Ding

**Affiliations:** 1 Prevention Research Collaboration, Sydney School of Public Health, Faculty of Medicine, The University of Sydney, Camperdown, New South Wales, Australia; 2 Sydney Nursing School, Charles Perkins Centre, The University of Sydney, Camperdown, New South Wales, Australia; Istituto Di Ricerche Farmacologiche Mario Negri, ITALY

## Abstract

**Background:**

There is growing evidence that breastfeeding has short- and long-term cardiovascular health benefits for mothers. The objectives of this systematic review were to examine the association between breastfeeding and maternal cardiovascular risk factors and outcomes that have not previously been synthesized systematically, including metabolic syndrome, hypertension and cardiovascular disease.

**Methods and findings:**

This systematic review meets PRISMA guidelines. The MEDLINE, EMBASE and CINAHL databases were systematically searched for relevant publications of any study design from the earliest publication date to March 2016. The reference lists from selected articles were reviewed, and forward and backward referencing were conducted. The methodological quality of reviewed articles was appraised using validated checklists.

Twenty-one studies meeting the inclusion criteria examined the association between self-reported breastfeeding and one or more of the following outcomes: metabolic syndrome/metabolic risk factors (n = 10), inflammatory markers/adipokines (n = 2), hypertension (n = 7), subclinical cardiovascular disease (n = 2), prevalence/incidence of cardiovascular disease (n = 3) and cardiovascular disease mortality (n = 2). Overall, 19 studies (10 cross-sectional/retrospective, 9 prospective) reported significant protective effects of breastfeeding, nine studies (3 cross-sectional/retrospective, 5 prospective, 1 cluster randomized controlled trial) reported non-significant findings and none reported detrimental effects of breastfeeding. In most studies reporting significant associations, breastfeeding remained associated with both short- and long-term maternal cardiovascular health risk factors/outcomes, even after covariate adjustment. Findings from several studies suggested that the effects of breastfeeding may diminish with age and a dose-response association between breastfeeding and several metabolic risk factors. However, further longitudinal studies, including studies that measure exclusive breastfeeding, are needed to confirm these findings.

**Conclusions:**

The evidence from this review suggests that breastfeeding is associated with cardiovascular health benefits. However, results should be interpreted with caution as the evidence gathered for each individual outcome was limited by the small number of observational studies. Additional prospective studies are needed.

**PROSPERO registration number:**

CRD42016047766.

## Introduction

Cardiovascular disease is the leading cause of death among women globally [1] and lifestyle-related factors play a key role in its prevention. Considerable attention has been given to more conventional risk factors such as obesity, physical inactivity and an unhealthy diet. However, other modifiable behaviours, such as breastfeeding, should be considered and incorporated in the development of potential strategies to prevent cardiovascular disease.

While the importance of breastfeeding is well recognized for infant and child health, there is growing interest in maternal health outcomes. Breastfeeding has favourable short-term effects on maternal metabolic health, including lipid homeostasis [2–4], glucose homeostasis and insulin sensitivity [5,6]. Evidence from observational studies is accumulating for an association between breastfeeding and longer-term maternal cardiovascular risk factors such as hypertension [7,8], type 2 diabetes [9,10], obesity [11], and metabolic syndrome [MS] [12]. Breastfeeding has also been linked to cardiovascular disease incidence [13] and mortality [14].

To date, there have been several systematic reviews examining the association between breastfeeding and cardiovascular risk factors such as postpartum weight change, body composition and type 2 diabetes [15–18]. Findings from these reviews suggest that breastfeeding may be associated with a reduced risk of type 2 diabetes [16,18] while the associations with postpartum weight change and body composition are unclear [15,17,18]. To our knowledge, the evidence for an association between breastfeeding and other cardiovascular risk factors and outcomes such as MS, hypertension and cardiovascular disease, has not been systematically summarized. With growing evidence suggesting that breastfeeding has both short- and long-term effects on maternal cardiovascular health outcomes, it is important to evaluate whether breastfeeding can be a modifiable risk factor for cardiovascular disease in parous women and whether lactation has long-term beneficial effects for maternal cardiovascular health.

Therefore, the objectives of this systematic review were to summarize the relationship of breastfeeding with maternal cardiovascular risk factors and outcomes that have not previously been reviewed systematically and to synthesize the findings that have been recently evaluated systematically. Reviewing this evidence systematically can provide valuable information for future guidelines and strategies for cardiovascular disease prevention.

## Methods

Details of the protocol for this systematic review were registered with the International Prospective Register of Systematic Reviews (PROSPERO; registration number CRD42016047766) and can be accessed at http://www.crd.york.ac.uk/PROSPERO/display_record.asp?ID=CRD42016047766. This systematic review meets Preferred Reporting Items for Systematic Reviews and Meta-Analyses (PRISMA) guidelines (S1 Checklist) [19].

### Search strategy

The electronic databases MEDLINE, EMBASE and CINAHL were searched for relevant publications, from the earliest publication date to March 2016, using multiple subject headings and text words in combination (S1 Table). Additional articles were identified through backward and forward reference searching. Authors of published conference abstracts were contacted to identify any corresponding full text publications. Only full text publications of studies on humans and published in English were considered.

### Inclusion criteria and study selection

Articles of any study design (e.g. cross-sectional/retrospective, prospective cohort, cluster randomized controlled trial [RCT]) were included in this systematic review if they investigated the association of breastfeeding with any maternal cardiovascular risk factor and/or cardiovascular outcome of a biological nature. Possible cardiovascular risk factors included: weight change, body mass index (BMI), waist circumference, body composition (e.g. visceral adiposity), hypertension, type 2 diabetes mellitus, hyperlipidemia, MS/risk factors and inflammatory markers. Studies on lifestyle risk factors, such as smoking, physical inactivity and an unhealthy diet, were not considered. Cardiovascular outcomes included subclinical and clinical cardiovascular disease prevalence, incidence and mortality. All study time periods, definitions of breastfeeding (whether exclusive or complemented by other foods) and studies that involved women with any menopausal status were accepted. Studies were excluded if they had a small sample size (defined arbitrarily as <100 participants) and if they examined risk factors/outcomes relating only to: the breastfed child, cancer, and pregnancy complications (e.g. gestational diabetes mellitus, pre-eclampsia and pre-term delivery). Two reviewers (BN and KJ) independently screened the titles and abstracts of retrieved articles to assess study eligibility. Any disagreement or uncertainty was resolved through discussion. The same reviewers reviewed the full text articles that met the inclusion criteria or with uncertain eligibility. Any disagreement was resolved by consensus. Although systematic reviews were not included among the selected studies, recent systematic reviews were identified and summarized for several outcomes of interest (postpartum weight change, body composition and type 2 diabetes) [15–18]. Studies involving maternal outcomes of interest that had not been previously systematically assessed were reviewed (MS/metabolic risk factors, hypertension, inflammatory markers, adipokines, subclinical cardiovascular disease, and cardiovascular disease prevalence, incidence and mortality).

### Quality assessment and data extraction

One reviewer (BN) assessed the methodological quality of cross-sectional/retrospective and prospective cohort studies by using an adapted 15-item checklist derived from checklists for the reporting of observational studies (S2 Table) [20]. The single cluster RCT trial was appraised against a quality assessment checklist based on a tool developed by the Cochrane collaboration for assessing risk of bias in randomized studies [21] and relating to the following criteria: random sequence generation, allocation concealment, blinding of participants and personnel, blinding of outcome assessment, completeness of outcome data, accurate outcome reporting and other sources of bias addressed. For each study, an overall study quality rating was allocated based on the total number of individual criteria met or addressed. Studies were rated as: “low quality” if ≤1/3 of individual criteria were met, “medium quality” if >1/3-≤2/3 of individual criteria were met and “high quality” if >2/3 of criteria were met. Five articles (~25%) were randomly selected from the included studies and independently appraised by the second reviewer (KJ). The overall agreement rate between both authors for the quality rating of these five articles was 100%.

The following data were extracted from each article: study design, country in which the study was conducted, cohort/study designation, sample size, brief participant description, age range, mean follow-up or period, type of outcome measure(s), breastfeeding comparison categories, effect sizes (most commonly reported as odds ratios or relative risks with 95% confidence intervals) and covariates adjusted for. The expected direction of each association was hypothesized based on existing literature and coded as: + (significant association in the hypothesized direction),–(significant association not in the hypothesized direction), 0 (non-significant association). Due to the heterogeneous nature of the studies and limited number of studies for each outcome of interest, only a qualitative analysis of included studies was conducted.

### Breastfeeding terms and categories

In this review, the terms breastfeeding and lactation are used interchangeably. Breastfeeding was self-reported in all included studies. Lactation history refers to any reported history (usually ≥1 month) of breastfeeding (ever vs. never). Lactation duration is the reported length of time a woman breastfed a child. Exclusive lactation duration is the length of time a woman exclusively breastfed a child before introducing complementary foods. Lifetime lactation duration is the cumulative amount of time a woman reportedly breastfed across all pregnancies and average lactation duration is the average amount of time a woman breastfed each child.

## Results

### Selection of studies

**Fig 1:**
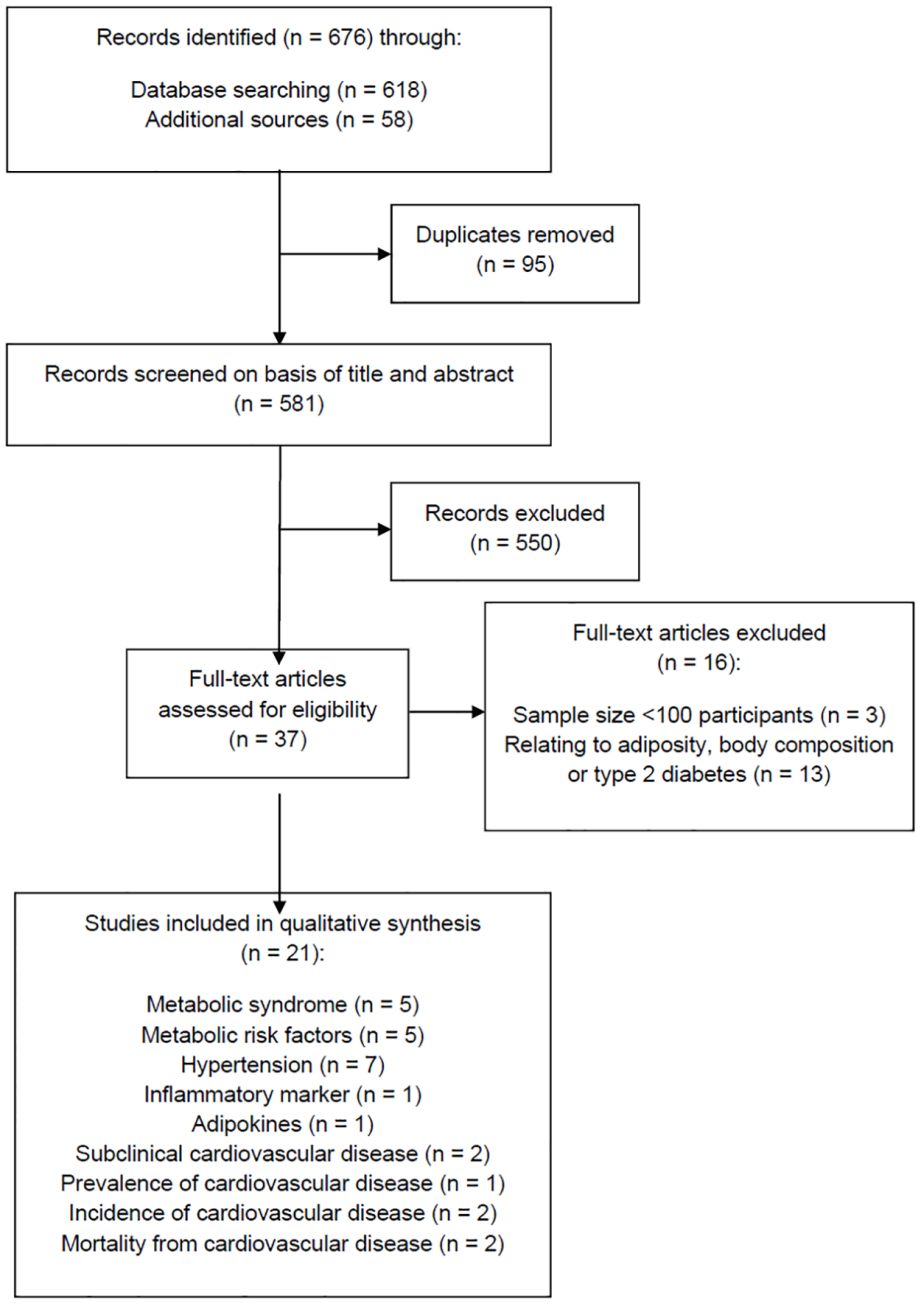
Selection of articles for systematic review.

The study selection process is shown in Fig 1. The literature searches yielded 581 unique citations, of which 37 were identified as potentially relevant. Following full text review, 16 studies were excluded based on small sample size or if they related to outcomes of interest that had been recently reviewed in retrieved systematic reviews (i.e., adiposity, body composition, and type 2 diabetes). Twenty-one articles examining the association between breastfeeding and one or more of the following outcomes were included for review: MS/metabolic risk factors (n = 10), hypertension (n = 7), inflammatory markers/adipokines (n = 2), subclinical cardiovascular disease (n = 2), prevalence/incidence of cardiovascular disease (n = 3) and cardiovascular disease mortality (n = 2).

### Critical appraisal

Out of 21 included papers, 16 (76%) were rated as high quality and 5 (24%) as medium quality (S3 and S4 Tables). Although most quality assessment criteria were adequately addressed, many observational studies failed to describe the reliability (n = 15) and/or validity (n = 12) of the breastfeeding measure and the number of participants with missing data for the exposure/outcome of interest (n = 6).

### Study characteristics

Tables 1–5 provide details of reviewed studies. Nearly all papers (95%) were published in the last decade. Of the 21 included studies, 9 were cross-sectional/retrospective [22–30], 10 were prospective [7,8,12–14,31–35], 1 reported both cross-sectional/retrospective and prospective data [36], and 1 was a cluster RCT [37]. More than half of the studies were conducted in the United States (US) (n = 11), with the remaining conducted in Europe (n = 4), Asia (n = 5), and Australia (n = 1). Sample sizes ranged from 297 to 267,400 participants (median = 6,914) and the age of participants varied between 18 and 89 years of age. In prospective studies, participants were followed up between 3 and 20 years. Breastfeeding was assessed mainly by self-administered questionnaires (16/21 studies) and also by interviewer-administered questionnaires (6/21 studies). Study outcomes were mostly measured, although several were self-reported.

**Table 1:** Summary of included studies with metabolic syndrome as the outcome.

First author (year)	Country and cohort designation	Participants	Mean follow-up or period (years)	Outcome assessment	Breastfeeding comparison categories^a^	Adjusted OR or RR (95% CI) by lactation history/ duration	Covariates
Cross-sectional/retrospective studies							
Cho et al. (2009) [24]	Korea, Korean National Health and Nutrition Examination Survey	892 post-menopausal women; 43–89 years	N/A	Measured; Prevalent MS	Ever (≥1 month) vs. never	1.20 (0.65, 2.20)	Age, marital status, SES, smoking, alcohol, PA, BMI
Ram et al. (2008) [22]	US, Study of Women’s Health Across the Nation (SWAN)	2,516 parous, pre-menopausal women; 42–52 years	N/A	Measured; Prevalent MS	Ever vs. never Per year lifetime	0.77 (0.62, 0.96) 0.88 (0.77, 0.99)	Study site, age, ethnicity, SES, smoking, PA, caloric intake, high school BMI, parity
Cohen et al. (2006) [23]	US, Third National Health and Nutrition Examination Survey (NHANES III)	4,699 non-pregnant, parous women; ≥20 years	N/A	Measured; Prevalent MS	Ever (≥1 month) vs. never	1.02 (0.78, 1.34)	Age, ethnicity, SES, smoking, alcohol, PA, BMI, OC, HRT
Prospective studies							
Ramezani Tehrani et al. (2014) [31]	Iran, Tehran Lipid and Glucose Study (TLGS)	925 women without prevalent MS at baseline; 15–50 years at baseline	9	Measured; Incident MS	Lifetime lactation duration; Never 1–6 months 7–12 months 13–23 months ≥24 months	1.5 (0.7, 3.0) 1.8 (0.7, 4.1) 1.5 (0.7, 3.2) 1.8 (1.0, 3.4) Reference	Age, PA, caloric intake, BMI, parity
Gunderson et al. (2010) [12]	US, Coronary Artery Risk Develop-ment in Young Adults (CARDIA) Study	620 nulliparous women without prevalent MS at baseline; delivered ≥1 singleton live birth during the follow-up period; 18–30 years at baseline	20	Measured; Incident MS	Lifetime lactation duration; 0–1 month >1–5 months 6–9 months >9 months P-trend	Reference 0.61 (0.36, 1.05) 0.52 (0.29, 0.93) 0.44 (0.23, 0.84) 0.03	Study centre, age, ethnicity, SES, smoking, PA, BMI, MS components^b^, parity

Abbreviations: BMI = body mass index, CI = confidence interval, HRT = hormonal replacement therapy, MS = Metabolic Syndrome, N/A = not applicable, OC = oral contraceptives, OR = odds ratio, PA = physical activity, RR = relative risk, SES = socioeconomic status, US = United States.

^a^ For the assessment of breastfeeding, self-reported measures included lactation history: defined as ever breastfeeding; lactation duration: length of time a woman breastfed a child; exclusive lactation duration: length of time a woman exclusively breastfed a child before introducing complementary foods; lifetime lactation duration: cumulative amount of time a woman breastfed across all pregnancies and average lactation duration: lifetime lactation duration divided by the total number of children. For lactation history, the reference category is the second category mentioned (e.g. for ever vs. never, never is the reference category).

^b^ For this study, MS components related to waist circumference measure, fasting triglyceride levels, high-density lipoprotein cholesterol levels, systolic or diastolic blood pressure or treatment with antihypertensive medication, and fasting glucose levels or treatment with diabetes medication.

**Table 2:** Summary of included studies with metabolic risk factors as the outcome.

First author (year)	Country and cohort designation	Participants	Mean follow-up or period (years)	Outcome assessment	Breastfeeding comparison categories^a^	Adjusted OR or RR (95% CI) by lactation history/ duration	Covariates
Cross-sectional studies							
Henriques et al. (2015)^b^ [25]	Portugal, Birth cohort generation XXI	1,847 mothers from public hospital maternity clinics; normal BMI: mean age (SD) = 34.4 (5.2); overweight BMI: mean age (SD) = 35.2 (5.2); obese BMI: mean age (SD): 35.3 (5.3)	N/A Assessed 4 years postpar-tum	Healthy metabolic phenotype (outcomes measured or self-reported medications) defined as the absence of HT, diabetes, dyslipidemia, CRP≤3mg/L and <2nd tertile of HOMA-IR	Lactation duration; Normal BMI Ow metab healthy: Never ≤26 weeks >26 weeks Ow metab not healthy: Never ≤26 weeks >26 weeks Ob metab healthy: Never ≤26 weeks >26 weeks Ob metab not healthy: Never ≤26 weeks >26 weeks	Reference Reference 0.84 (0.39, 1.82) 1.10 (0.50, 2.40) Reference 0.58 (0.33, 1.00) 0.64 (0.37, 1.12) Reference 0.65 (0.18, 2.33) 0.85 (0.23, 3.08) Reference 0.44 (0.26, 0.74) 0.39 (0.23, 0.68)	Age, family history of CVD/cardiometabolic risk factors, PA, OC
Natland et al. (2012) [26] ≤50 years	Norway, Nord-Trøndelag Health Survey (HUNT2)	21,368 non-pregnant, parous women without prevalent MI, stroke, angina pectoris or diabetes prior to the first birth; not currently/ previously taking anti-hypertensive medication for BP data analysis; and with TG levels <4.5 mmol/L for LDL-C analyses; 20–85 years	N/A	Measured; metabolic risk factors WC; non-fasting: serum levels of TG, total chol, HDL-C, LDL-C and blood glucose	Lifetime lactation duration; Never 1–6 months 7–12 months 13–23 months ≥24 months	Lifetime lactation duration was inversely associated with WC, TG, total chol, LDL-C, and HDL-C (all P-trends<0.001 except for HDL-C P-trend = 0.008). P-trend for blood glucose was not significant. The estimates were attenuated after further adjustment for BMI (for all lipids; no association remained for HDL-C) but remained similar after adjustment for time since last birth.	Age, marital status, SES, smoking, PA, time since last meal (for serum lipids and blood glucose), parity
Natland et al. (2012) [26] >50 years	-	-	-	-	-	Significant association with WC only (p-trend = 0.03)	-
Schwarz et al. (2009) [36]	US, Women’s Health Initiative (WHI)	139,681 parous, postmenopau-sal women with ≥1 live birth; 50–79 years	N/A	Measured and/or self-reported (use of chol-lowering medication) hyperlipidemia	Lifetime lactation duration: Never 1–6 months 7–12 months 13–23 months ≥24 months P-trend Never ≥12 months	Reference 0.93 (0.89, 0.97) 0.88 (0.83, 0.94) 0.81 (0.76, 0.87) 0.80 (0.73, 0.87) <0.0001 Reference 0.81, p<0.001	Age, ethnicity, SES, family history of diabetes/MI/ stroke, smoking, PA, dietary intake, use of aspirin/multivitamin, BMI, parity, HRT
Prospective studies							
Stuebe et al. (2010) [33]	US, Project Viva	570 women with a singleton pregnancy; <22 weeks gestation at baseline; mean age (SD) at 3 years postpartum: no lactation: 36.0 (4.8); >0–<3 months lactation: 36.8 (6.0); 3–<6 months: 37.2 (5.3); 6–<12 months: 38.2 (4.6); ≥12 months: 38.8 (5.0)	3 years postpar-tum	Measured; BMI, WC and metabolic markers: HbA1c, SHBG, fasting insulin, glucose, HOMA-IR, total chol, LDL-C, HDL-C, TG 175 subjects had fasting blood samples	Lactation duration: Never >0-<3 months 3-<6 months 6-<12 months ≥12 months Exclusive lactation duration: Never >0-<1 months 1-<3 months 3-<6 months ≥6 months	No significant associations between either lactation duration or exclusive lactation duration and outcome measures. Adjustment for BMI before pregnancy eliminated all unadjusted associations with HOMA_IR, fasting insulin, SHBG and 3-year postpartum WC.	Family history of type 2 diabetes, smoking, PA, dietary intake, intention to lose weight, self-reported weight at 12 months, pre-pregnancy BMI, gestational weight gain, gestational glucose tolerance, parity, OC
Gunderson et al. (2007) [32]	US, Coronary Artery Risk Develop-ment in Young Adults (CARDIA) Study	1,051 non-pregnant women or who delivered 1 singleton live birth during the 3-year interval, without prevalent MS at baseline; 24–42 years at baseline (year 7)	3 (interval between years 7–10 follow-up)	Measured; mean change in metabolic risk factors: fasting plasma glucose, insulin, HOMA-IR, LDL-C, total chol, HDL-C, TG, BP, weight, WC	Non-pregnant, no lactation, and lactated and weaned (post-weaning) groups. Post-weaning group also dichotomised based on lactation duration: <3 and ≥3 months	Both the no lactation and post-weaning groups had greater adjusted mean gains in WC and decrements in HDL-C than non-pregnant women (all p<0.001). LDL-C (p<0.05) and fasting insulin (p = 0.06) increased more for the no lactation group compared to the other two groups. ≥3 months lactation was associated with a smaller decrement in HDL-C than <3 months (p<0.01).	Baseline age, ethnicity, SES, smoking, BMI, time since weaning to year 10 examination (for analyses within post-weaning group only), parity, OC

Abbreviations: BMI = body mass index, BP = blood pressure, chol = cholesterol, CI = confidence interval, CRP = C-reactive protein, CVD = cardiovascular disease, HbA1c = hemoglobin A1c, HDL-C = high-density lipoprotein cholesterol, HOMA-IR = homeostasis model assessment of insulin resistance, HRT = hormonal replacement therapy, HT = hypertension, LDL-C = low-density lipoprotein cholesterol, metab = metabolically, MI = myocardial infarction, N/A = not applicable, Ob = obese, OC = oral contraceptives, OR = Odds Ratio, Ow = overweight, PA = physical activity, RR = relative risk, SD = standard deviation, SES = socioeconomic status, SHBG = sex hormone-binding globulin, TG = triglycerides, US = United States, WC = waist circumference.

^a^ For the assessment of breastfeeding, self-reported measures included lactation history: defined as ever breastfeeding; lactation duration: length of time a woman breastfed a child; exclusive lactation duration: length of time a woman exclusively breastfed a child before introducing complementary foods; lifetime lactation duration: cumulative amount of time a woman breastfed across all pregnancies and average lactation duration: lifetime lactation duration divided by the total number of children. For lactation history, the reference category is the second category mentioned (e.g. for ever vs. never, never is the reference category).

^b^ In this study, women who breastfed their child >26 weeks were less likely to be obese and “metabolically unhealthy” (defined as the presence of HT, diabetes, dyslipidemia, CRP>3mg/L and >2nd tertile of HOMA-IR).

**Table 3:** Summary of included studies with hypertension as the outcome.

First author (year)	Country and cohort designation	Participants	Mean follow-up or period (years)	Outcome assessment	Breastfeeding comparison categories^a^	Adjusted OR or RR (95% CI) by lactation history/ duration	Covariates
Cross-sectional/retrospective studies							
Zhang et al. (2015) [27]	China	9,128 parous women with only one lifetime birth; 40–81 years	N/A	Measured or self-reported (previous physician diagnosis of HT or current use of anti-hypertensive medication) HT	Never vs. ever P-trend Lactation duration; Never >0–6 months >6–12 months >12 months P-trend	1.18 (1.05, 1.32) 0.01 Reference 0.87 (0.76, 0.99) 0.83 (0.68, 1.0) 0.79 (0.65, 0.97) 0.04	Age, SES, family history of HT, smoking, alcohol, BMI, postpartum BMI, WHR, age of menarche, age of child-bearing, menopausal status, OC
Lupton et al. (2013) [28]	Australia, 45 and Up Study	74,785 women who had never given birth or who gave birth between 18–45 years of age and who were not diagnosed with HT during pregnancy; ≥45 years	N/A	Self-reported HT (having been treated in the last month)	Nulliparous Parous, never Parous, ever	Reference 1.06 (0.95–1.18)^b^ 0.89 (0.82–0.97)^b^	Age, country of origin, SES, family history of HT, smoking, alcohol, PA, BMI, OC, HRT
Lupton et al. (2013) [28] 45-<54 years	-	-	-	-	Lifetime lactation duration; Parous, never 1-<3 months 3-<6 months 6-<12 months 12-<18 months 18->24 months ≥24 months Average lactation duration; Parous, never 1-<3 months 3-<6 months 6-<12 months 12-<18 months ≥18 months	Reference 0.88 (0.63, 1.24)^b^ 0.87 (0.62, 1.20)^b^ 0.74 (0.55, 0.98)^b^ 0.71 (0.53, 0.95)^b^ 0.57 (0.41, 0.79)^b^ 0.58 (0.44, 0.77)^b^ Reference 0.87 (0.66, 1.16)^b^ 0.76 (0.57, 1.0)^b^ 0.61 (0.47, 0.80)^b^ 0.60 (0.44, 0.81)^b^ 0.62 (0.42, 0.91)^b^	-
Lupton et al. (2013) [28] 54-<64 years	-	-	-	-	Lifetime lactation duration; Parous, never 1-<3 months 3-<6 months 6-<12 months 12-<18 months 18->24 months ≥24 months Average lactation duration; Parous, never 1-<3 months 3-<6 months 6-<12 months 12-<18 months ≥18 months	Reference 0.97 (0.78, 1.20)^b^ 0.84 (0.69, 1.02)^b^ 0.81 (0.68, 0.96)^b^ 0.78 (0.64, 0.94)^b^ 0.71 (0.57, 0.89)^b^ 0.60 (0.50, 0.73)^b^ Reference 0.90 (0.76, 1.06)^b^ 0.81 (0.69, 0.97)^b^ 0.71 (0.60, 0.84)^b^ 0.64 (0.51, 0.81)^b^ 0.54 (0.39, 0.76)^b^	-
Lupton et al. (2013) [28] ≥64 years	-	-	-	-	Lifetime lactation duration; Parous, never 1-<3 months 3-<6 months 6-<12 months 12-<18 months 18->24 months ≥24 months Average lactation duration; Parous, never 1-<3 months 3-<6 months 6-<12 months 12-<18 months ≥18 months	Reference 1.08 (0.89, 1.31)^b^ 1.11 (0.94, 1.31)^b^ 1.04 (0.90, 1.20)^b^ 1.06 (0.91, 1.24)^b^ 1.07 (0.90, 1.28)^b^ 1.03 (0.88, 1.22)^b^ Reference 1.06 (0.92, 1.22)^b^ 1.04 (0.91, 1.20)^b^ 1.12 (0.97, 1.29)^b^ 0.87 (0.65, 1.18)^b^ 0.36 (0.17, 0.84)^b^	-
Natland et al. (2012) [26] ≤50 years	Norway, Nord-Trøndelag Health Survey (HUNT2)	21,368 non-pregnant, parous women without prevalent MI, stroke, angina pectoris or diabetes prior to the first birth; not currently/ previously taking anti-hypertensive medication; 20–85 years	N/A	Measured or self-reported (current use of anti-hypertensive medication) HT	Lifetime lactation duration; Never 1–6 months 7–12 months 13–23 months ≥24 months P-trend	1.88 (1.41, 2.51) 1.24 (1.03, 1.49) 1.16 (0.98, 1.37) 1.03 (0.88, 1.21) Reference <0.001 The estimates were attenuated after adjustment for BMI and time since last birth.	Age, marital status, SES, smoking, PA, time since last meal (for serum lipids and blood glucose), parity
Natland et al. (2012) [26] >50 years	-	-	-	-	Lifetime lactation duration; Never 1–6 months 7–12 months 13–23 months ≥24 months P-trend	1.26 (0.96, 1.65) 0.88 (0.75, 1.02) 0.93 (0.80, 1.07) 0.89 (0.78, 1.01) Reference 0.944	
Schwarz et al. (2009) [36]	US, Women’s Health Initiative (WHI)	139,681 postmenopau-sal women with ≥1 live birth; 50–79 years	N/A	Measured or self-reported (history of treated HT) HT	Lifetime lactation duration: Never 1–6 months 7–12 months 13–23 months ≥24 months P-trend Never ≥12 months	Reference 0.95 (0.92, 0.98) 0.88 (0.84, 0.92) 0.89 (0.84, 0.93) 0.87 (0.82, 0.93) <0.0001 Reference 0.88, p<0.001	Age, ethnicity, SES, family history of diabetes/MI/ stroke, smoking, PA, dietary intake, use of aspirin/multivitamin, BMI, parity, HRT
Prospective studies							
Stuebe et al. (2011) [7]	US, US Nurses' Health Study II	55,636 parous women without prevalent HT, diabetes, CVD, hyperlipidemia or cancer at baseline; 25–42 years at baseline	1991–2005	Self-reported; incidence of HT (previously diagnosed by a physician excluding during pregnancy)	Lactation duration for the first child: Never >0–3 months >3-<6 months 6-<9 months 9-<12 months ≥12 months P-trend Exclusive lactation duration: Never Breastfed, never exclusively >0–3 months >3-<6 months ≥6 months P-trend Average lactation duration: Never >0–3 months >3-<6 months 6-<9 months 9-<12 months ≥12 months P-trend Average exclusive lactation duration: Never Breastfed, never exclusively >0–3 months >3-<6 months ≥6 months P-trend	1.27 (1.18, 1.36) 1.29 (1.20, 1.39) 1.16 (1.08, 1.25) 1.11 (1.03, 1.19) 1.03 (0.95, 1.11) Reference <0.001 1.29 (1.20, 1.39) 1.11 (1.03, 1.19) 1.08 (0.99, 1.18) 1.03 (0.95, 1.12) Reference <0.001 1.22 (1.13, 1.32) 1.21 (1.12, 1.30) 1.19 (1.11, 1.28) 1.09 (1.02, 1.18) 1.07 (0.99, 1.17) Reference <0.001 1.16 (1.05, 1.27) 1.14 (1.04, 1.24) 1.08 (0.99, 1.18) 1.04 (0.95, 1.13) Reference <0.001	Race, family history of HT, history of pregnancy complications, smoking, alcohol, vigorous PA, DASH diet score quintile, BMI at age 18 years, nonnarcotic analgesic use, year of first birth, OC
Lee et al. (2005) [8]	Korea, Korean Women's Cohort (KWC) Study	177,749 pre-menopausal women without prevalent HT at baseline; 20–59 years at baseline	6	Measured or self-reported (current use of anti-hypertensive medication) HT	Ever vs. never Lifetime lactation duration: Never 1–6 months 7–12 months 13–18 months 19–24 months >24 months Average lactation duration: Never 1–3 months 4–6 months 7–9 months 10–12 months >12 months Normal weight and lactation Normal weight, no lactation Obese and lactation Obese, no lactation	0.92 (0.90, 0.96) Reference 0.90 (0.87, 0.93) 0.92 (0.87, 0.98) 0.93 (0.86, 0.99) 1.0 (0.91, 1.11) 1.06 (0.99, 1.14) Reference 0.90 (0.87, 0.94) 0.90 (0.85, 0.96) 0.93 (0.86, 0.99) 1.0 (0.95, 1.10) 1.02 (0.96, 1.08) Reference 1.06 (1.02, 1.11) 1.65 (1.58, 1.72) 1.85 (1.75, 1.90)	Age, smoking, PA, BMI>23.05, age at menarche, age at first pregnancy, number of children, OC
Cluster randomized controlled trial							
Oken et al. (2013) [37]	Belarus, Promotion of Breastfeeding Intervention Trial (PROBIT)	9,383 breastfeeding mothers who delivered a healthy singleton live birth at ≥37 weeks gestation; 31 units of medical care randomised to breastfeeding promotion intervention group or usual care; mean baseline age (SD): intervention group: 25.0 (5.0) and control group: 25.1 (4.9)	11.5 years postpar-tum	Measured or self-reported (previous diagnosis of HT or current use of anti-hypertensive medication) HT	Observational analyses regardless of treatment allocation: Lactation duration; <3 months ≥3-<6 months ≥6-<9 months ≥9-<12 months ≥12 months P-trend Exclusive lactation duration: <3 months ≥3-<6 months ≥6 months P-trend	No significant difference between the intervention and control groups for SBP (adjusted mean difference [95% CI] = 20.23 [22.71, 2.25]), DBP (adjusted mean difference [95% CI] = 20.74 [22.02, 0.53]), any HT (AOR [95% CI] = 0.88 [0.65, 1.19]) or diagnosed HT (AOR [95% CI] = 0.81 [0.45, 1.44]) Baseline and cluster-adjusted mean difference; Reference 0.10 (-0.67, 0.87) -1.19 (-2.12, -0.25) -0.71 (-1.85, 0.42) -0.57 (-1.48, 0.33) 0.05 Reference -0.39 (-1.16, 0.38) 0.40 (-1.16, 1.97) 0.75	Study site, polyclinic location, age, SES, smoking during pregnancy, number of children in the household

Abbreviations: BMI = body mass index, CI = confidence interval, CVD = cardiovascular disease, DASH = Dietary Approaches to Stop Hypertension, DBP = diastolic blood pressure, HRT = hormonal replacement therapy, HT = hypertension, MI = myocardial infarction, N/A, not applicable; OC = oral contraceptives, OR = odds ratio, PA = physical activity, RR = relative risk, SD = standard deviation, SES = socioeconomic status, SBP = systolic blood pressure, US = United States, WHR = waist-to-hip ratio.

^a^ For the assessment of breastfeeding, self-reported measures included lactation history: defined as ever breastfeeding; lactation duration: length of time a woman breastfed a child; exclusive lactation duration: length of time a woman exclusively breastfed a child before introducing complementary foods; lifetime lactation duration: cumulative amount of time a woman breastfed across all pregnancies and average lactation duration: lifetime lactation duration divided by the total number of children. For lactation history, the reference category is the second category mentioned (e.g. for ever vs. never, never is the reference category).

^b^ Odds ratio with 99% CI presented for this paper.

**Table 4:** Summary of included studies with inflammatory markers, adipokines and subclinical cardiovascular disease as the outcomes.

First author (year)	Country and cohort designation	Participants	Mean follow-up or period (years)	Outcome assessment	Breastfeeding comparison categories^a^	Adjusted OR or RR (95% CI) by lactation history/ duration	Covariates
Cross-sectional/retrospective studies							
McClure et al. (2012) [29]	US, Women and Infant Study of Healthy Hearts (WISH)	569 premenopausal women who delivered a singleton live birth, following a pregnancy without complications; mean age (SD) = 35.6 (7) for women who breastfed any child <3 months; mean age (SD) = 39.6 (6) for women who breastfed ≥3 months	N/A 4–12 years after delivery	Measured subclinical CVD: carotid artery intima-media thickness, lumen diameter, adventitial diameter and carotid-femoral pulse wave velocity	Lactation duration for each child: Never breastfed Breastfed any child <3 months Breastfed each child ≥3 months Never breastfed Breastfed any child <3 months Breastfed each child ≥3 months Never breastfed Breastfed any child <3 months Breastfed each child ≥3 months Never breastfed Breastfed any child <3 months Breastfed each child ≥3 months	Lumen diameter 0.13 (0.04, 0.22) 0.11 (0.002, 0.22) Reference Adventitial diameter 0.12 (0.02, 0.22) 0.10 (-0.02, 0.21) Reference Intima-media thickness 0.79 (0.41, 1.54) 0.51 (0.24, 1.09) Reference Carotid-femoral pulse wave velocity 0.21 (-0.10, 0.52) 0.02 (-0.34, 0.37) Reference	Age, ethnicity, SES, family history of diabetes/MI/stroke, smoking, PA, vitamin supplementation, early adult BMI, current BMI, SBP, total chol, HDL-C, TG, CRP, glucose, insulin, optimism, anxiety, max. gestational weight gain, birth outcome, gestational age, infant birth weight, additional preterm births, years since last birth, parity
Schwarz et al. (2010) [30]	US, Study of Women’s Health Across the Nation (SWAN)	297 women without prevalent CVD at baseline who delivered at least 1 singleton live birth; 45–58 years	N/A	Measured subclinical CVD: coronary and aortic calcification, carotid adventitial diameter, intima–media thickness and carotid plaque	Lactation duration for each child: Never breastfed Breastfed any child <3 months Breastfed each child ≥3 months Never breastfed Breastfed any child <3 months Breastfed each child ≥3 months Never breastfed Breastfed any child <3 months Breastfed each child ≥3 months Never breastfed Breastfed any child <3 months Breastfed each child ≥3 months Never breastfed Breastfed any child <3 months Breastfed each child ≥3 months	Aortic calcification Reference 0.34 (0.09, 1.28) 0.19 (0.05, 0.68) Coronary calcification Reference 0.96 (0.28, 3.27) 0.43 (0.13, 1.49) Carotid plaque Reference 0.75 (0.18, 3.23) 0.45 (0.11, 1.84) Adventitial diameter Reference -0.12 (-0.35, 0.11) -0.04 (-0.26, 0.18) Intima-media thickness Reference 0.79 (0.27, 2.32) 0.93 (0.33, 2.67)	Study site, age, ethnicity, SES, family history of diabetes/MI/stroke, smoking, PA, dietary intake, vitamin supplementation, BMI, SBP, TG, total chol, HDL, CRP, glucose, insulin, perceived stress, depressed mood, menopausal status, parity
Prospective studies							
Stuebe et al. (2010) [33]	US, Project Viva	570 women with a singleton pregnancy; <22 weeks gestation at baseline; mean age (SD) at 3 years postpartum: no lactation: 36.0 (4.8); >0–<3 months lactation: 36.8 (6.0); 3–<6 months: 37.2 (5.3); 6–<12 months: 38.2 (4.6); ≥12 months: 38.8 (5.0)	3 years post-partum	Measured inflammato-ry markers: CRP and IL6 175 subjects had fasting blood samples	Lactation duration: Never >0-<3 months 3-<6 months 6-<12 months ≥12 months Exclusive lactation duration: Never >0-<1 months 1-<3 months 3-<6 months ≥6 months	No significant associations between either lactation duration or exclusive lactation duration and inflammatory markers. Adjustment for BMI before pregnancy eliminated unadjusted association with CRP.	Family history of type 2 diabetes, smoking, PA, dietary intake, intention to lose weight, self-reported weight at 12 months, pre-pregnancy BMI, gestational weight gain, gestational glucose tolerance, parity, OC
Stuebe et al. (2011) [34]	US, Project Viva	570 women with a singleton pregnancy; <22 weeks gestation at baseline; mean age (SD) at 3 years postpartum: no lactation: 36.0 (4.8); >0–<3 months lactation: 36.8 (6.0); 3–<6 months: 37.2 (5.3); 6–<12 months: 38.2 (4.6); ≥12 months: 38.8 (5.0)	3 years post-partum	Measured adipokines: leptin, adiponectin, ghrelin, peptide YY 175 subjects had fasting blood samples	Lactation duration: Never >0-<3 months 3-<6 months 6-<12 months ≥12 months Exclusive lactation duration: Never >0-<1 months 1-<3 months 3-<6 months ≥6 months	Lactation duration was associated with ghrelin (predicted mean = 749.5 for none vs. 852.9 pg/ml for ≥12 months lactation; p = 0.05) and peptide YY levels (predicted geometric mean = 55 for none vs. 63.4 pg/ml for ≥12 months lactation; p = 0.03). Lactation duration was not associated with leptin levels after adjustment for pre-pregnancy BMI. Exclusive lactation duration was associated with ghrelin (predicted mean = 790.6 for never exclusively breastfeeding vs. 1,008.1 pg/ml for 6 months exclusive breastfeeding; p<0.01). Non-linear association between lactation duration/exclusive lactation duration with adiponectin.	Age, race, family history of type 2 diabetes, smoking status, pre-pregnancy BMI, gestational weight gain, gestational glucose tolerance, parity; additional adjustment for lactation duration after introduction of complementary foods

Abbreviations: BMI = body mass index, chol = cholesterol, CI = confidence interval, CRP = C-reactive protein, CVD = cardiovascular disease, HDL-C = high-density lipoprotein cholesterol, IL-6 = interleukin 6, max = maximum, MI = myocardial infarction, N/A = not applicable; OC = oral contraceptives, OR = odds ratio, PA = physical activity, RR = relative risk, SBP = systolic blood pressure, SD = standard deviation, SES = socioeconomic status, TG = triglycerides, US = United States.

^a^ For the assessment of breastfeeding, self-reported measures included lactation history: defined as ever breastfeeding; lactation duration: length of time a woman breastfed a child; exclusive lactation duration: length of time a woman exclusively breastfed a child before introducing complementary foods.

**Table 5:** Summary of included studies with cardiovascular disease as the outcome.

First author (year)	Country and cohort designation	Participants	Mean follow-up or period (years)	Outcome assessment	Breastfeeding comparison categories^a^	Adjusted OR or RR (95% CI) by lactation history/ duration	Covariates
Cross-sectional/retrospective studies							
Schwarz et al. (2009) [36]	US, Women's Health Initiative (WHI)	139,681 parous, postmenopau-sal women with ≥1 live birth; 50–79 years	N/A	Self-reported; prevalence of CVD (MI, angina, CHF, peripheral arterial disease, revascularisation, stroke)	Lifetime lactation duration: Never 1–6 months 7–12 months 13–23 months ≥24 months P-trend Never ≥13 months	Reference 1.03 (0.98, 1.09) 0.95 (0.88, 1.02) 0.93 (0.85, 1.01) 0.89 (0.80, 0.98) 0.005 Reference 0.91 (0.85, 0.98), P = 0.008	Age, ethnicity, SES, family history of diabetes/MI/ stroke, smoking, PA, dietary intake, use of aspirin/multivitamin, BMI, parity, HRT
Prospective studies							
Stuebe et al. (2009) [13]	US, Nurses' Health Study	89,326 parous women without a history of MI, angina or coronary artery bypass graft; 40–65 years	1986–2002	Self-reported incidence of MI (confirmed by physician review of medical records)	Lifetime lactation duration: None >0–3 months >3–6 months >6–11 months >11–23 months >23 months P-trend	Reference (0.91, 1.11)^b^ (0.88, 1.14)^b^ (0.88, 1.18)^b^ 0.93 (0.8, 1.07)^b^ 0.77 (0.62, 0.94)^b^ 0.02	Age, parental history of MI before age 60 years, smoking, alcohol, PA, dietary intake, use of aspirin/multivitamins, BMI at age 18 years, birth weight of subject, history of stillbirth, menopausal status, parity, HRT
Stuebe et al. (2009) [13] Birth in the last 30 years	-	-	-	-	Lifetime lactation duration; None >0–3 months >3–6 months >6–11 months >11–23 months >23 months P-trend	Reference 0.94 (0.79, 1.12)^b^ 0.98 (0.80, 1.21)^b^ 0.96 (0.76, 1.21)^b^ 0.89 (0.71, 1.10)^b^ 0.66 (0.49, 0.89)^b^ 0.02	-
Stuebe et al. (2009) [13] No birth in the last 30 years	-	-	-	-	Lifetime lactation duration; None >0–3 months >3–6 months >6–11 months >11–23 months >23 months P-trend	Reference 1.04 (0.92, 1.18) 1.02 (0.86, 1.21) 1.02 (0.84, 1.24) 0.95 (0.78, 1.15) 0.90 (0.67, 1.19) 0.33	-
Schwarz et al. (2009) [36]	US, Women's Health Initiative (WHI)	139,681 parous, postmenopau-sal women with ≥1 live birth; 50–79 years	7.9	Self-reported incidence of CVD (CHD, stroke, CHF, angina, peripheral vascular disease, carotid artery disease, and coronary revascularization) validated by physician adjudication of medical records	Lifetime lactation duration: Never 1–6 months 7–12 months 13–23 months ≥24 months P-trend	Reference (0.98, 1.08)^b^ 0.97 (0.90, 1.04)^b^ 0.98 (0.91, 1.05)^b^ 0.93 (0.85, 1.02)^b^ 0.12	Age, ethnicity, SES, family history of diabetes/MI/stroke, smoking, dietary intake, use of aspirin/multivitamin, PA, BMI, parity, HRT
Gallagher et al. (2011) [35]	China	259,494 non-smoking female workers employed in the textile industry; 30->60 years	1989–2000	Measured; mortality from IHD, ischaemic stroke and haemorrhagic stroke, based on a death registry	Lactation duration; IHD: Never (parous) <6 months 7–12 months 13–24 months 25–36 months 37–48 months ≥49 months Ischaemic stroke: Never (parous) <6 months 7–12 months 13–24 months 25–36 months 37–48 months ≥49 months Haemorrhagic stroke: Never (parous) <6 months 7–12 months 13–24 months 25–36 months 37–48 months ≥49 months	Reference 0.70 (0.42, 1.16)^b^ 0.50 (0.33, 0.76)^b^ 0.67 (0.46, 0.97)^b^ 0.53 (0.36, 0.79)^b^ 0.71 (0.48, 1.06)^b^ 0.78 (0.53, 1.14)^b^ Reference 1.02 (0.63, 1.66)^b^ 1.05 (0.72, 1.54)^b^ 0.90 (0.62, 1.31)^b^ 1.15 (0.79, 1.67)^b^ 1.21 (0.83, 1.77)^b^ 1.20 (0.84, 1.72)^b^ Reference 0.84 (0.63, 1.12)^b^ 0.98 (0.79, 1.22)^b^ 1.01 (0.82, 1.24)^b^ 0.88 (0.71, 1.09)^b^ 1.02 (0.82, 1.28)^b^ 1.05 (0.84, 1.30)^b^	Age, number of live births
Natland Fagerhaug et al. (2013) [14] <65 years	Norway, Nord-Trøndelag Health Survey (HUNT2)	21,889 non-pregnant, parous women without prevalent MI, stroke, angina pectoris or diabetes prior to the first birth; 30–85 years	15	Measured; mortality from CVD, based on a death registry	Ever lactated Never lactated Nulliparous Lifetime lactation duration: None 7–12 months ≥24 months P-linear trend	Reference 2.86 (1.51, 5.39)^b^ 0.41 (0.16, 1.04)^b^ 2.77 (1.28, 5.99)^b^ 0.55 (0.27, 1.09)^b^ Reference 0.8	Marital status, SES, smoking, PA, BMI, TG, total chol, SBP, DBP, use of antihypertensive medication, diabetes, parity
Natland Fagerhaug et al. (2013) [14] ≥65 years	-	-	-	-	Ever lactated Never lactated Nulliparous Lifetime lactation duration:	Reference 1.11 (0.77, 1.69)^b^ 1.20 (1.0. 1.44)^b^ No clear associations (hazard ratios not reported in the study).	-

Abbreviations: BMI = body mass index, CHD = coronary heart disease, CHF = congestive heart failure, chol = cholesterol, CI = confidence interval, CVD = cardiovascular disease, DBP = diastolic blood pressure, HRT = hormone replacement therapy, IHD = ischaemic heart disease, MI = myocardial infarction, N/A = not applicable, OR = odds ratio, RR = relative risk, SBP = systolic blood pressure, SES = socioeconomic status, TG = triglycerides, US = United States.

^a^ For the assessment of breastfeeding, self-reported measures included lactation history: defined as ever breastfeeding; lactation duration: length of time a woman breastfed a child; lifetime lactation duration: cumulative amount of time a woman breastfed across all pregnancies.

^b^ Hazard ratios with 95% CI presented for this paper.

### Association between breastfeeding and maternal cardiovascular risk factors and outcomes

#### Weight change/body composition

A 2014 systematic review by Neville et al., based on 37 prospective studies and eight retrospective studies, assessed the relationship between breastfeeding and changes in postpartum weight or body composition in mothers ≤2 years postpartum [17]. Most studies found little or no association between breastfeeding and either change in postpartum weight or maternal body composition.

In 2015, a systematic review conducted by Chowdury et al. [15] updated the review on postpartum weight change by Neville et al. [17] with five additional studies. The authors concluded that there were no clear associations between breastfeeding and postpartum weight change, with factors such as age, gestational weight gain and pre-pregnancy weight possibly confounding these relationships.

#### Type 2 diabetes

In a 2014 review and meta-analysis based on six cohort studies, the longest duration of lifetime lactation was associated with a 32% reduction in relative risk of type 2 diabetes compared with the shortest duration [16]. This finding was in line with a later review of the same primary studies [15] and an earlier systematic review [18].

#### MS

MS is a cluster of conditions which can increase the risk for diabetes and CVD. Table 1 describes studies with MS as the outcome. Five studies examined the association between breastfeeding and MS [12,22–24,31]. Studies adhered to the National Cholesterol Education Program Adult Treatment Panel III criteria [38] to define MS, which is based on the presence of three of more of the following risk determinants: abdominal obesity, elevated triglyceride levels, reduced high-density lipoprotein cholesterol levels, elevated blood pressure, and elevated fasting glucose levels [38]. The findings from the three cross-sectional studies, conducted among women of different age categories, were mixed [22–24]. One study from the US found a significant protective association of both lactation history and lifetime lactation duration with MS in a dose-response manner in middle-aged, parous premenopausal women from various ethnic backgrounds [22]. Another US study reported a significant association between lactation history and the prevalence of MS in parous women aged ≥20 years. However, this association was no longer significant after additional adjustment for BMI [23]. The third study did not find any association between lactation history and the prevalence of the MS in postmenopausal Korean women [24].

Both prospective studies found significant protective effects of lifetime lactation duration on incident MS [12,31]. One study following Iranian women over 9 years observed a significant association between 13–23 months lifetime lactation duration and higher incidence of MS compared with ≥24 months [31]. The other study showed that lifetime lactation duration of 6–9 and >9 months was significantly associated with a lower incidence of MS compared with 0–1 month lactation, among American women of reproductive age followed over a 20-year period [12].

#### Metabolic risk factors

Table 2 provides a summary of studies with metabolic risk factors as the outcomes. Five studies (three cross-sectional/retrospective, two prospective) assessed the relation between breastfeeding and metabolic risk factors [25,26,32,33,36]. All three cross-sectional/retrospective studies reported a protective association of breastfeeding with metabolic risk factors [25,26,36]. One study involving Portuguese mothers who were examined at 4 years postpartum, showed that women who breastfed their child for >26 weeks were less likely to be obese and have and an adverse metabolic profile. However, there was no association between breastfeeding and excessive weight associated with a healthy metabolic profile [25]. Another cross-sectional/retrospective study found that lifetime lactation duration is associated in a dose-response fashion with a more favorable cardiovascular risk profile, including lipids, in a large sample of Norwegian mothers later in life [26]. Similarly, the third cross-sectional/retrospective study found a dose-response relationship between lifetime lactation duration and hyperlipidemia in a large sample of US mothers [36].

The findings from relatively small prospective studies were mixed [32,33]. One US prospective study examining 3-year changes in metabolic risk factors from pre-pregnancy to post-weaning showed that breastfeeding is associated with a more favorable metabolic risk profile in the postpartum period [32]. In contrast, the other US prospective study did not find any association between lactation duration and metabolic risk at 3 years postpartum after adjusting for pre-pregnancy BMI [33].

#### Hypertension

Table 3 describes studies with hypertension as the outcome. Seven studies examined the association between breastfeeding and hypertension [7,8,26–28,36,37]. Breastfeeding was associated with lower odds of hypertension in all four cross-sectional/retrospective studies [26–28,36]. Both lactation history and duration were associated with reduced odds of hypertension in middle-aged and older Chinese mothers [27]. Similarly, in a large sample of Australian women aged ≥45 years, lactation history was protective compared to parous women who never breastfed or nulliparous women [28]. Lifetime lactation duration of >6 months, or >3 months/child, was significantly associated with lower odds of hypertension, in women aged 45–64 years compared with parous women who did not breastfeed. The odds of hypertension decreased with longer breastfeeding durations and were mostly not significant in women ≥64 years. In another cross-sectional/retrospective study from Norway, while there were no clear associations in mothers ≥50 years, mothers aged <50 years who had never lactated had higher odds of hypertension than those who had lactated ≥24 months in their lifetime [26]. In contrast, lifetime lactation duration was significantly associated with lower odds of hypertension in postmenopausal US women ≥50 years [36].

Breastfeeding also appeared to be protective in two large prospective studies [7,8]. In a US cohort of parous women, those who did not breastfeed were more likely to develop hypertension compared with those who breastfed their first child for ≥12 months or exclusively breastfed their first child for ≥6 months [7]. Lactation history, lifetime lactation duration between 1–18 months and average lactation duration between 1–9 months were also protective among a large cohort of premenopausal Korean women followed for six years [8]. In a large cluster RCT from Belarus, despite greater breastfeeding duration and exclusivity achieved among breastfeeding mothers randomized to a breastfeeding promotion intervention compared to usual care, there was no significant difference in blood pressure between mothers receiving the intervention and those allocated to the usual care group. However, this was not an RCT of breastfeeding per se but of factors aimed at promoting breastfeeding behaviour. In addition, a marginally significant association was found between lactation duration and lower blood pressure at 11.5 years postpartum in observational analyses in the same sample regardless of treatment allocation [37].

#### Inflammatory markers and adipokines

Table 4 provides a summary of studies with inflammatory markers, adipokines and subclinical cardiovascular disease as the outcomes. Only two papers from the same sample were identified [33,34]. In one paper, two inflammatory markers: C-reactive protein (CRP), commonly associated with cardiovascular health outcomes [39], and interleukin-6, a proinflammatory cytokine that induces the hepatic production of CRP [40], were examined, but neither was significantly related to lactation duration at 3 years postpartum among 175 women with fasting blood samples after adjusting for pre-pregnancy BMI [33]. The other paper [34] examined adipokines, which are cytokines secreted by adipose tissue that are involved in inflammatory responses and associated with metabolic disease risk [41]. At 3 years postpartum, longer lactation duration was associated with higher levels of ghrelin and peptide YY, both involved in appetite regulation and associated with reduced risk of metabolic disease [34].

#### Subclinical cardiovascular disease

Early physiologic changes in vascular health such as calcified atherosclerotic plaques and increases in carotid adventitial diameter can be detected and identify patients at increased risk of future cardiovascular events [42–44]. Two cross-sectional/retrospective studies from the US assessed the relationship between breastfeeding and subclinical cardiovascular disease [29,30]. Both studies have found non-breastfeeding mothers to be at increased risk of vascular changes associated with subsequent cardiovascular disease. Among premenopausal women assessed 4 to 12 years after delivery, mothers who never breastfed had larger carotid artery lumen and adventitial diameters, which are indicative of poorer cardiovascular health status, compared with mothers who breastfed all of their children for at least 3 months [29]. In another study involving an older sample of women between 45 to 58 years of age, the association between lactation and an increased adventitial diameter was not significant after adjustment for confounders. However, aortic calcification remained significantly associated with lactation duration [30]. McClure et al. [29] suggest that differences in the significance of findings between breastfeeding and adventitial diameter could be due to the younger age group and shorter time since pregnancy in their study.

#### Cardiovascular disease

Table 5 describes studies with cardiovascular disease as the outcome. A few studies investigated the relationship between breastfeeding and cardiovascular disease, and found protective effects of breastfeeding [13,36]. One US study examined both the self-reported prevalence and incidence (confirmed by physician adjudication of medical records) of cardiovascular disease in a large sample of parous, postmenopausal women [36]. In that study, increasing lifetime lactation duration was significantly associated with a lower prevalence of cardiovascular disease, compared with never breastfeeding. In particular, women who breastfed ≥13 months in their lifetime and women aged 50–59 years who had breastfed ≥7 months, were less likely to have prevalent cardiovascular disease. Although women aged 60–69 years with 13–23 months of lifetime lactation had lower odds of prevalent cardiovascular disease than similar-aged women who had never breastfed, there were no significant associations observed in women aged 70–79 years [36].

In that same study, lifetime lactation duration was not associated with incident cardiovascular disease in the overall sample followed for 7.9 years [36]. However, compared to similar-aged women who never breastfed, significant cardiovascular benefits were seen in women in the younger age group, but not in the older age groups. In another large US study involving middle-aged and elderly women, women with a lifetime lactation duration ≥12 months had a reduced risk of incident myocardial infarction (confirmed by physician review of medical records) compared with parous women who had never breastfed [13]. A stronger inverse association was observed for women with ≥23 months of lifetime lactation and for those with a birth in the last 30 years.

Two prospective studies assessed the association between breastfeeding and cardiovascular disease mortality ascertained from death registries [14,35]. Among a large sample of Chinese non-smoking textile workers followed between 1989 and 2000, lactation duration was not significantly associated with mortality from ischaemic or haemorrhagic stroke [35]. However, compared to parous women who never breastfed, women who breastfed appeared to have a lower risk of mortality from ischaemic heart disease. In another study from Norway, mothers aged <65 years that never breastfed had nearly three times the risk of death from cardiovascular disease over 15 years compared with mothers with a lifetime lactation duration ≥24 months [14]. There was evidence for a U-shaped association with women who breastfed 7–12 months having almost half the risk of women who breastfed ≥24 months in their lifetime. There were no significant associations in women 65 years and over.

#### Overall pattern of associations

The pattern of associations across different outcomes is summarized in Table 6. Overall, 19 studies (10 cross-sectional/retrospective, 9 prospective) reported significant protective effects of breastfeeding, nine studies (3 cross-sectional/retrospective, 5 prospective, 1 cluster RCT) reported non-significant findings and none reported detrimental effects of breastfeeding. Ten out of thirteen associations reported in cross-sectional/retrospective studies suggested that breastfeeding was associated with significant cardiovascular benefits. Although the evidence was less convincing, nine out of fifteen associations reported in prospective studies also indicated beneficial effects of breastfeeding. Out of all cardiovascular risk factors and outcomes considered, the evidence for significant protective effects of breastfeeding was most convincing for hypertension, although the evidence was mainly based on cross-sectional/retrospective studies. Three-quarters of high quality studies [7, 12, 13, 14, 22, 26, 28, 29, 30, 32, 34, 36] and 80% of medium quality studies [8, 27, 31, 35] reported significant protective effects of breastfeeding.

**Table 6:** Summary of expected direction of associations^a^ between breastfeeding and cardiovascular risk factors/outcomes.

	References	Number of cross-sectional/retrospective studies			Number of prospective studies/cluster randomized controlled trial		
		+	0	-	+	0	-
*Cardiovascular risk factors*							
Metabolic Syndrome	[12,22–24,31]	1	2		2		
Metabolic risk factors	[25,26,32,33,36]	2			1	1	
Hypertension	[7,8,26–28,36,37]	4			2	1	
Inflammatory markers	[33]					1	
Adipokines	[34]				1	1	
Subclinical cardiovascular disease	[29,30]	2	1				
*Cardiovascular outcomes*							
Prevalence/incidence of cardiovascular disease	[13,36]	1			1	1	
Cardiovascular disease mortality	[14,35]				2	1	

^a^ The expected direction of each association was hypothesized based on existing literature and coded as: + (significant association in the hypothesized direction),–(significant association not in the hypothesized direction), 0 (non-significant association).

## Discussion

This review synthesized the current evidence on the associations between breastfeeding and cardiovascular risk factors and outcomes, including MS, metabolic risk factors, hypertension, inflammatory markers, adipokines, subclinical cardiovascular disease and cardiovascular disease. Nearly all included studies were published in the last decade highlighting the rising interest in the maternal health benefits of breastfeeding. Overall, most studies reported significant protective effects of breastfeeding, several reported non-significant findings while there were no studies that reported detrimental effects of breastfeeding. The cardiovascular benefits of breastfeeding were present in most studies even after adjustment for multiple covariates, including socio-demographic, lifestyle factors (e.g. smoking, physical activity, dietary intake), BMI and parity. In addition, findings from included studies indicate that breastfeeding has favorable short-term and long-term cardiovascular health outcomes. Altogether, the evidence from medium-high quality studies suggests that breastfeeding is associated with several cardiovascular health benefits that can extend to later life, and supports health promotion strategies and interventions to increase breastfeeding. Notwithstanding, findings from this review should be interpreted with caution as the evidence gathered for each individual outcome is limited by the small number of observational studies. In particular, additional prospective studies of larger samples are needed.

### Breastfeeding intensity and duration

For optimal child and maternal health benefits, the World Health Organization recommends exclusive breastfeeding for the first 6 months of life followed by two years or more of breastfeeding supplemented by complementary foods [45]. With the exception of four studies [7,33,34,37], most studies did not distinguish between exclusive breastfeeding, a measure of breastfeeding intensity, and breastfeeding supplemented by other foods. Several studies compared exclusive breastfeeding for ≥6 months with shorter durations of exclusive breastfeeding or non-exclusive breastfeeding, and found inconsistent associations with a range of cardiovascular outcomes [7,33,34,37]. Overall, additional studies that explore the association between exclusive breastfeeding and a range of short- and long-term cardiovascular health outcomes are needed.

Breastfeeding appears to be a protective factor for several maternal cardiovascular risk factors and outcomes, with evidence suggesting that increased duration may be associated with further benefits. Based on evidence from both cross-sectional/retrospective and prospective studies, benefits were reported for ≥24 months of lifetime lactation for most outcomes including metabolic risk factors [26,36], hypertension [28], the prevalence [36] and incidence of cardiovascular disease [13], and mortality from cardiovascular disease [14].

### Dose-response

A dose-response relationship has been suggested between breastfeeding and various cardiovascular outcomes including MS, several metabolic risk factors, hypertension and the prevalence of cardiovascular disease. A cross-sectional/retrospective study among parous pre-menopausal women found a dose-response association between lifetime lactation duration and MS [22]. However, this relationship was modified by parity and protective effects of breastfeeding were no longer observed after four births. A prospective study also found dose-response effects of lifetime lactation duration up to >9 months on incident MS developed over a 20-year period [12]. In two large cross-sectional/retrospective studies involving parous women <50 years [26] and postmenopausal women [36], inverse dose-response associations were observed between lifetime lactation duration up to ≥24 months and several maternal cardiovascular risk factors including lipid levels [26,36], blood pressure [26,36], and the prevalence of cardiovascular disease [36].

Although findings from these studies suggest that there is a dose-response relationship between breastfeeding and metabolic risk factors, additional evidence is needed, particularly from longitudinal studies. The possibility of a U-shaped relationship between breastfeeding and cardiovascular mortality [14] should also be further investigated.

### Short vs long-term outcomes

A previous systematic review has evaluated the relationship between breastfeeding and postpartum weight change and has found inconclusive evidence [17]. In our systematic review, lactation was associated with maternal improvements in metabolic risk factors from preconception to post-weaning in one prospective study [32] while no association was detected in another prospective study at 3 years postpartum [33]. However, an association between breastfeeding and adipokine levels at 3 years postpartum was reported in the latter cohort [34].

Meanwhile, evidence from cross-sectional/retrospective studies among middle-aged and older women suggests that breastfeeding may have protective effects in later life against hypertension [22,28], metabolic risk [26,36] and cardiovascular disease [36].

Findings from prospective studies suggest that breastfeeding may be protective against the incidence of: MS among young and middle-aged women followed for 9 [31] or 20 years [12], hypertension among young and middle-aged women after 6 years of follow-up [7,8], cardiovascular disease among middle-aged [13] and older women [36] followed between 8–12 years, mortality from ischaemic heart disease over a 10 year period [35] and mortality from cardiovascular disease in women of various ages followed for 15 years [14].

#### Age, time since last birth and menopause

Findings from one cross-sectional/retrospective study [26] and a few prospective studies [14,28,36] suggest that the benefits from breastfeeding may diminish with age. In a large cross-sectional Norwegian study, significant associations between lifetime lactation duration and cardiovascular risk factors were observed in parous women <50 years. However, there were no clear associations in women >50 years [26]. Among three prospective studies, there were unclear associations with hypertension [28], the incidence of cardiovascular disease [36], or cardiovascular disease mortality [14] in women >60 [36] or >65 years of age [14,28]. Time since last birth may also have an effect on the association between breastfeeding and cardiovascular outcomes [13,26,36]. Among Norwegian mothers <50 years, the association between lifetime lactation duration and hypertension was attenuated after adjustment for time since last birth, while the association with metabolic risk factors remained similar [26]. In a large prospective cohort study, there was a stronger association between breastfeeding and incident myocardial infarction for women who gave birth in last the 30 years compared to women who had not [13]. Menopausal status may also influence the risk of cardiovascular disease. Among a large cohort of postmenopausal women, increasing lifetime lactation duration was associated with a lower prevalence of hypertension, diabetes, hyperlipidemia and cardiovascular disease [36]. There was also a significant association between breastfeeding and the incidence of cardiovascular disease among women between 50–59 years of age, but not among women >60 years [36]. Whether older age, increasing time since last birth and menopause attenuate the association between breastfeeding and cardiovascular risk factors and outcomes requires further investigation.

### Potential mechanisms

Breastfeeding increases metabolic expenditure approximately by 480 calories/day [46]. Although the effects of breastfeeding on postpartum weight change remains inconclusive [15,17], breastfeeding may lower cardiovascular risk by mobilizing fat stores accumulated during pregnancy. Breastfeeding may also have favorable effects on glucose metabolism, glycemic control and lipid metabolism [2–6]. The “reset hypothesis” in which breastfeeding “resets” maternal metabolism after pregnancy by reversing visceral fat accumulation and increases in insulin resistance, lipid and triglyceride levels has been proposed [47]. Hormones associated with breastfeeding such as prolactin and oxytocin may also exert effects on maternal blood pressure [48–50]. In addition, oxytocin may promote mother-child attachment and lead to reduced stress levels.

### Methodological considerations

Several methodological issues should be considered in interpreting these findings. Observational studies are subject to residual confounding. Unmeasured confounders could include health-enhancing behaviors of breastfeeding mothers that distinguish them from non-breastfeeding mothers, and factors that influence breastfeeding initiation and duration such as pre-pregnancy BMI and pre-existing metabolic risk factors [51–53]. Breastfeeding measures were self-reported and recall bias may have led to misclassification of a woman’s lactation history such as under- or over reporting of breastfeeding duration [54]. However, maternal recall of breastfeeding has been shown to be valid and reliable [55]. Differences in findings could be due to a number of factors that vary between studies including sample characteristics (e.g. country, setting), breastfeeding comparison categories, covariate adjustment and follow-up periods for prospective studies. Most included studies did not assess the exclusivity of breastfeeding, a measure of breastfeeding intensity. Temporal relationships could not be established from cross-sectional studies.

### Strengths and limitations

The strengths of this review include systematic literature search, data extraction and summarization, an evaluation of the quality of included studies using established checklists, a range of maternal cardiovascular risk factors and outcomes examined, the inclusion of various breastfeeding comparison categories, the assessment of evidence relating to exclusive breastfeeding and dose-response relationship between breastfeeding and maternal outcomes, as well as a systematic and detailed approach in reporting findings. The limitations of this systematic review reflects limitations of the existing literature, such as a small number of prospective studies for each outcome of interest and methodological issues described above. Although the search terms used were comprehensive, there is a possibility that relevant studies were not identified by this systematic review.

### Conclusion

Overall, the evidence from this systematic review suggests that breastfeeding is associated with maternal cardiovascular health benefits that extend from child-bearing years to later life. However, additional longitudinal research is needed to investigate the association between breastfeeding and specific cardiovascular risk factors and outcomes and to further inform the evidence base.

## Supporting information

S1 Checklist PRISMA 2009 checklist. (DOC)

S1 Table Search strategy used for MEDLINE, which was then adapted for EMBASE and CINAHL. (DOCX)

S2 Table Quality assessment criteria adapted from a 15-item checklist used by Van Uffelen et al. [20]. (DOCX)

S3 Table Critical appraisal of included cross-sectional and prospective studies based on 15 quality assessment criteria. (DOCX)

S4 Table Critical appraisal of single cluster randomized controlled trial [37] based on quality assessment checklist developed by the Cochrane collaboration for assessing risk of bias in randomized studies [21]. (DOCX)
