# Supplementary material for: Breastfeeding and maternal cardiovascular risk factors and outcomes: A systematic review
Source: PLoS One. 2017 Nov 29;12(11):e0187923. doi: 10.1371/journal.pone.0187923 (PMC5706676; doi:10.1371/journal.pone.0187923)
Supplement: S1 Table — (DOCX) [file pone.0187923.s002.docx]

**S1 Table. Search strategy used for MEDLINE, which was then adapted for EMBASE and CINAHL.**

1. Breast Feeding (as a subject heading)
2. Lactation (as a subject heading)
3. Milk, Human (as a subject heading)
4. Breast fed (as a text word)
5. Breastfe* (as a text word)
6. Lactat* (as a text word)
7. Breast milk (as a text word)
8. 1 or 2 or 3 or 4 or 5 or 6 or 7
9. Maternal Health (subject heading)
10. Maternal health (as a text word)
11. Exp Women’s Health (subject heading)
12. Reproductive history (subject heading)
13. (wom#n adj2 health) (as a text word)
14. (mother* adj2 health) (as a text word)
15. 9 or 10 or 11 or 12 or 13 or 14
16. Cardiovascular diseases (subject heading)
17. Hypertension (subject heading)
18. Risk factors (subject heading)
19. Coronary Artery Disease (subject heading)
20. Obesity (subject heading)
21. Diabetes Mellitus, Type 2 (subject heading)
22. Myocardial ischemia (subject heading)
23. Ischemic heart disease (as a text word)
24. Ischaemic heart disease (as a text word)
25. 16 or 17 or 18 or 19 or 20 or 21 or 22 or 23 or 24
26. 8 and 15 and 25
27. Limit 26 to (English language and female and humans)
