# Supplementary material for: Breastfeeding and maternal cardiovascular risk factors and outcomes: A systematic review
Source: PLoS One. 2017 Nov 29;12(11):e0187923. doi: 10.1371/journal.pone.0187923 (PMC5706676; doi:10.1371/journal.pone.0187923)
Supplement: S2 Table — (DOCX) [file pone.0187923.s003.docx]

**S2 Table. Quality assessment criteria adapted from a 15-item checklist used by Van Uffelen et al. [31]**

| **Item** | **Criteria** | **Description** |
| --- | --- | --- |
| 1 | Objectives | Are the objectives or hypotheses of the research described in the paper stated? |
| 2 | Study design | Is the study design presented? |
| 3 | Target population | Do the authors describe the target population they wanted to research? |
| 4 | Sample | Was a random sample of the target population taken? AND was the response rate 60% or more? |
| 5 | Sample | Is participant selection described? |
| 6 | Sample | Is participant recruitment described, or referred to? |
| 7 | Sample | Are the inclusion and/or exclusion criteria stated? |
| 8 | Sample | Is the study sample described? (minimum description=sample size, gender, age and an indicator of socio-economic status) |
| 9 | Sample | Are the numbers of participants at each stage of the study reported (Authors should report at least numbers eligible, numbers recruited, numbers with data at baseline, and numbers lost to follow-up) |
| 10 | Variables | Are the measures of breastfeeding and health outcome of interest described? |
| 11 | Data sources and collection | Do authors describe the source of their data (e.g., registry, health survey) AND did authors describe how the data were collected? (e.g., by mail) |
| 12 | Measurement | Was reliability of the measure(s) of breastfeeding mentioned or referred to? |
| 13 | Measurement | Was the validity of the measure(s) of breastfeeding mentioned or referred to? |
| 14 | Statistical methods | Were appropriate statistical methods used and described, including those for addressing confounders? |
| 15 | Statistical methods | Were the numbers/percentages of participants with missing data for breastfeeding and the health outcome indicated AND If more than 20% of data in the primary analyses were missing, were methods used to address missing data? |
