# Supplementary material for: Breastfeeding and maternal cardiovascular risk factors and outcomes: A systematic review
Source: PLoS One. 2017 Nov 29;12(11):e0187923. doi: 10.1371/journal.pone.0187923 (PMC5706676; doi:10.1371/journal.pone.0187923)
Supplement: S3 Table — (DOCX) [file pone.0187923.s004.docx]

| **First author (publication year) /**  **Quality assessment criteria addressed^a^** | **#1** | **#2** | **#3** | **#4** | **#5** | **#6** | **#7** | **#8** | **#9** | **#10** | **#11** | **#12** | **#13** | **#14** | **#15** | **Overall count^b^** | **Overall study quality rating^c^** |
| --- | --- | --- | --- | --- | --- | --- | --- | --- | --- | --- | --- | --- | --- | --- | --- | --- | --- |
| Cho (2009) [24] | Y | Y | Y | Y | Y | Y | Y | Y | Y | Y | U | Y | Y | Y | U | 13 Y, 2 U | High |
| Ram (2008) [22] | Y | Y | Y | U | Y | Y | Y | Y | Y | Y | Y | Y | Y | Y | Y | 14 Y, 1 U | High |
| Cohen (2006) [23] | Y | Y | Y | U | Y | Y | Y | Y | U | Y | Y | N | N | Y | U | 10 Y, 3 U, 2 N | Medium |
| Gunderson (2010) [12] | Y | Y | Y | Y | Y | Y | Y | Y | Y | Y | Y | N | N | Y | Y | 13 Y, 2 N | High |
| Gunderson (2007) [32] | Y | Y | Y | Y | Y | Y | Y | Y | Y | Y | Y | N | N | Y | Y | 13 Y, 2 N | High |
| Ramezani Tehrani (2014) [31] | Y | Y | Y | U | Y | Y | U | U | U | Y | Y | Y | Y | Y | N | 7 Y, 4 U, 1 N | Medium |
| Zhang (2015) [27] | Y | Y | Y | U | Y | N | N | Y | N | Y | Y | N | N | Y | U | 8 Y, 2 U, 5 N | Medium |
| Lupton (2013) [28] | Y | Y | Y | N | Y | Y | Y | Y | Y | Y | Y | N | N | Y | Y | 12 Y, 3 N | High |
| Lee (2005) [8] | Y | Y | Y | N | Y | Y | Y | U | U | Y | Y | N | N | Y | U | 9 Y, 3 U, 3 N | Medium |
| Stuebe (2011) [7] | Y | Y | Y | U | Y | N | Y | Y | Y | Y | Y | N | N | Y | Y | 11 Y, 1 U, 3 N | High |
| McClure (2012) [29] | Y | Y | Y | N | Y | Y | Y | Y | Y | Y | Y | N | N | Y | Y | 12 Y, 3 N | High |
| Schwarz (2010) [30] | Y | Y | Y | U | Y | U | Y | Y | Y | Y | Y | N | Y | Y | Y | 12 Y, 2 U, 1 N | High |
| Gallagher (2011) [35] | Y | Y | Y | N | Y | Y | U | Y | U | Y | Y | N | N | Y | U | 9 Y, 3 U, 3 N | Medium |
| Natland (2012) [26] | Y | Y | Y | N | Y | Y | Y | Y | Y | Y | Y | Y | Y | Y | Y | 14 Y, 1 N | High |
| Stuebe (2009) [13] | Y | Y | Y | N | Y | Y | Y | Y | Y | Y | Y | N | Y | Y | Y | 13 Y, 2 N | High |
| Henriques (2015) [25] | Y | Y | Y | N | Y | Y | Y | Y | Y | Y | Y | N | N | Y | Y | 12 Y, 2 N | High |
| Schwarz (2009) [36] | Y | Y | Y | N | Y | Y | Y | Y | Y | Y | Y | N | Y | Y | Y | 13 Y, 2 N | High |
| Stuebe (2011) [34] | Y | Y | Y | N | Y | Y | Y | U | Y | Y | Y | N | N | Y | Y | 11 Y, 1 U, 3 N | High |
| Natland (2013) [14] | Y | Y | Y | N | Y | Y | Y | Y | Y | Y | Y | Y | Y | Y | Y | 14 Y, 1 N | High |
| Stuebe (2010) [33] | Y | Y | Y | N | Y | Y | Y | U | Y | Y | Y | N | N | Y | Y | 11 Y, 1 U, 3 N | High |

**S3 Table. Critical appraisal of included cross-sectional/retrospective and prospective studies based on 15 quality assessment criteria criteria.**

Abbreviations: N=No, U=Unclear, Y=Yes.

^a^ Quality assessment criteria addressed were based on a checklist used by Van Uffelen et al. [20] and related to 15 criteria listed and described in S2 Table.

^b^ Each individual criteria was allocated a Y (yes) if it was met/addressed, N (no) if it was not met or U (unclear) if it was unclear whether the criteria was met. An overall count of the total number of individual criteria that were met, not met and that were unclear is provided.

^c^ An overall study quality rating was allocated based on the total number of criteria that were met (i.e., total number of “yes”). Studies were rated as: “low quality” if ≤1/3 of individual criteria were met, “medium quality” if >1/3-≤2/3 of individual criteria were met and “high quality” if >2/3 of criteria were met.
