# Supplementary material for: Breastfeeding and maternal cardiovascular risk factors and outcomes: A systematic review
Source: PLoS One. 2017 Nov 29;12(11):e0187923. doi: 10.1371/journal.pone.0187923 (PMC5706676; doi:10.1371/journal.pone.0187923)
Supplement: S4 Table — (DOCX) [file pone.0187923.s005.docx]

**S4 Table. Critical appraisal of single cluster randomized controlled trial [37] based on quality assessment checklist developed by the Cochrane Collaboration for assessing risk of bias in randomized studies [21].**

| **Quality assessment criteria** | **Was criteria met/addressed? ^a^** |
| --- | --- |
| **Random sequence generation** | Yes |
| **Allocation concealment** | Yes |
| **Blinding of participants and personnel** | No |
| **Blinding of outcome assessment** | No |
| **Completeness of outcome data** | Yes |
| **Accurate outcome reporting** | Yes |
| **Other sources of bias addressed** | Yes |
| **Overall count^b^** | 5 Yes, 2 No |
| **Overall study quality rating^c^** | High |

^a^ Each individual criteria was allocated a “yes” if it was met/addressed, “no” if it was not met or “unclear” if it was unclear whether the criteria was met.

^b^ An overall count of the total number of individual criteria that were met, not met and that were unclear is provided.

^c^ An overall study quality rating was allocated based on the total number of criteria that were met (i.e., total number of “yes”). Studies were rated as: “low quality” if ≤1/3 of individual criteria were met, “medium quality” if >1/3-≤2/3 of individual criteria were met and “high quality” if >2/3 of criteria were met.
